# Association between systemic immunity-inflammation index and sex hormones in children and adolescents aged 6–19

**DOI:** 10.3389/fendo.2024.1355738

**Published:** 2024-06-13

**Authors:** Zijun Gao, Ke Liu

**Affiliations:** Department of Laboratory Medicine, Traditional Chinese and Western Medicine Hospital of Wuhan, Tongji Medical College, Huazhong University of Science and Technology, Wuhan, China

**Keywords:** NHANES, systemic immune-inflammation index, sex hormones, children, adolescents

## Abstract

**Objectives:**

This study aimed to evaluate the relationship between systemic immune-inflammation index (SII) and sex hormones in children and adolescents aged 6–19 years.

**Methods:**

Data were obtained from the National Health and Nutrition Examination Survey (NHANES) conducted between 2013 and 2016. Inclusion criteria comprised subjects aged 6–19 years with complete data on both SII and sex hormones. We employed weighted multiple regression analysis and subgroup analytical methods to independently estimate the relationship between SII and sex hormones.

**Results:**

In this study, a total of 3767 participants were included, with an average age of 12.32 ± 3.95 years. Males constituted 50.54%, and females 49.46%. Among males, a statistically significant negative correlation emerged between SII and sex hormone-binding globulin (SHBG). Similarly, in the female population, SII exhibited a statistically significant negative correlation with total testosterone (TT), SHBG, and the Ratio of TT to estradiol, while maintaining a positive correlation with free androgen index (FAI). Subgroup analysis underscored variances in the association between sex hormones and SII within cohorts distinguished by pubertal status or different body mass index (BMI). In addition, the relationship between SII and estradiol exhibited nonlinearity. Employing a two-segment linear regression model, we identified an inverted U-shaped association between SII and estradiol, with an inflection point of 748.09 (1000cell/ml).

**Conclusion:**

Our findings suggest that SII may be an independent risk factor for changes in sex hormones in both male and female children and adolescents. More prospective and experimental studies should be conducted to validate our results and elucidate the underlying molecular pathways.

## 1 Introduction

Sex hormones play a crucial role in the growth and functioning of diverse tissues, influencing various aspects of health and disease. Testosterone (TT) is crucial for diagnosing hypogonadism and other androgen disorders (1). Low circulating testosterone levels in males lead to hypogonadism, which is a common issue in male reproductive health and sexual behavior (2). In addition to male reproductive health and sexual behavior, androgens are also essential for female reproduction. Female androgen imbalance leads to polycystic ovary syndrome. Moreover, androgens are associated with the development and progression of prostate cancer, breast cancer, and ovarian cancer (3). Estradiol is a common form of serum and tissue estrogen in both males and females, with most estrogen in the blood coming from the aromatization of testosterone in peripheral organs. It is responsible for the development of female secondary sexual characteristics and participates in the regulation of the female reproductive cycle. Moreover, for males, estrogen is indispensable for the proper development of cortical bone and the maintenance of healthy bone metabolism throughout the aging process (4, 5). Sex hormone-binding globulin (SHBG) is predominantly biosynthesized within hepatic tissues. Testosterone and estradiol circulate in the bloodstream, and some bind to SHBG. As a result, SHBG participates in regulating testosterone and estradiol (6, 7). Accumulated studies indicate that sex hormones play a pivotal role in shaping the development and growth of children and adolescents, orchestrating crucial physiological processes during this stage of life. Disturbances in sex hormone balance can potentially lead to a significant disease burden (8). Consequently, the management of sex hormones in the pediatric and adolescent population holds paramount significance.

The Systemic Immune-Inflammation Index (SII) is a novel and stable inflammation biomarker reflecting both local immune responses and systemic inflammation (9, 10). It is calculated by multiplying the platelet count by the ratio of the neutrophil to the lymphocyte count, providing a valuable measure of inflammation. Initially proposed for prognostic evaluation in hepatocellular carcinoma (11), it has subsequently undergone widespread scrutiny across various neoplastic disorders (10).

Research suggests that sex hormones, including female hormones such as estrogen and progesterone, and male hormones like testosterone directly affect the function of immune cells and inflammatory responses (12). Generally, androgens appear to have anti-inflammatory effects, while estrogens tend to have pro-inflammatory effects (13). Through ERα signal transduction, estradiol inhibits the differentiation of Th1 and Th17 cells, providing a protective mechanism against experimental inflammation (14). A reciprocal association has been elucidated between testicular hormones and inflammatory markers, such as IL-6 and TNF-α (15). However, the relationship between SII and sex hormones in children and adolescents is not yet clear.

Thus, we employed a substantial dataset drawn from children and adolescents from the National Health and Nutrition Examination Survey (NHANES) to investigate the association between SII and sex hormones. This exploration holds the potential to yield novel insights into sex hormone management within clinical practice.

## 2 Methods

### 2.1 Study design and population

This study utilized data from NHANES, a series of cross-sectional surveys conducted by the Centers for Disease Control and Prevention, to assess the nutritional and health status of the U.S. population (16). NHANES is an ongoing U.S. national cross-sectional survey using a complex multi-stage sampling strategy, repeated on a two-year cycle. All NHANES data are publicly available at https://www.cdc.gov/nchs/nhanes/.

**Figure 1:**
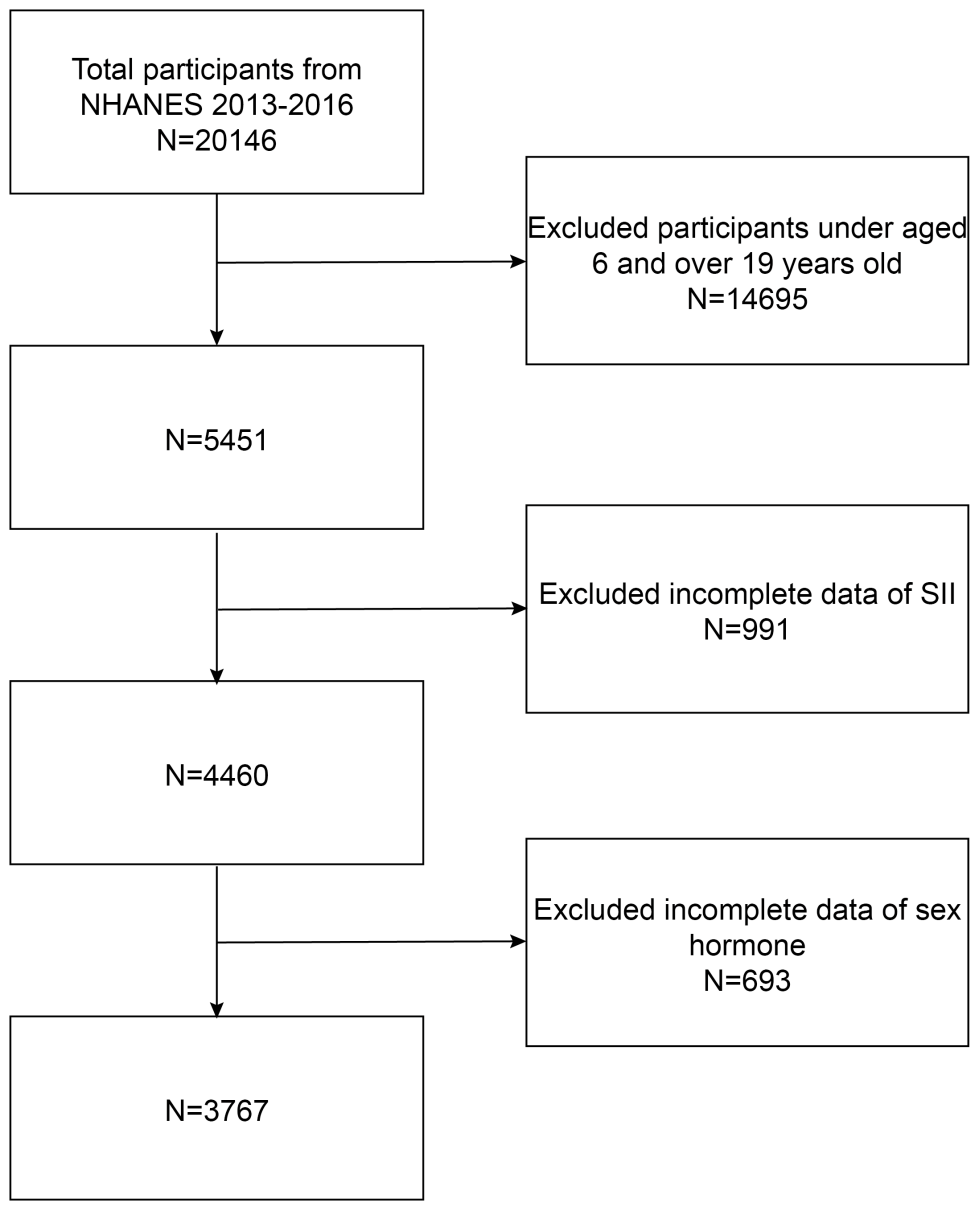
Flowchart of participant selection.

Considering that only the 2013–2014 and 2015–2016 survey cycles encompass complete data on SII and sex hormones, we elected to focus on these specific survey periods. Initially, our cohort comprised 20,146 participants. Following the exclusion of individuals aged less than 6 or greater than 19 years (14,695 individuals), those with missing SII data (991 individuals), and participants lacking data on sex hormones (639 individuals), we ultimately identified 3,767 eligible participants. The sample selection flowchart is depicted in **Figure 1**.

### 2.2 Exposure and outcome definition

The dependent variable in the current investigation was the systemic immunity-inflammation index, and the SII was designed as the exposure variable in our analysis. An automated hematology analysis device (Coulter^®^DxH 800 analyzer) was used to calculate whole blood counts of lymphocytes, neutrophils, and platelets, which were expressed as 10^3^ cells/mL. SII was calculated by platelet count × neutrophil count/lymphocyte count (11, 17).

Prior to transportation to the National Center for Environmental Health for testing, serum specimens were stored at -20°C. Serum TT and estradiol concentrations were determined using isotope dilution high-performance liquid chromatography tandem mass spectrometry (ID-LC-MS/MS). In contrast, SHBG concentrations were determined through the reaction of SHBG with immune antibodies and chemiluminescence measurements of reaction products using photomultiplier tubes. Furthermore, we also calculated the Free Androgen Index (FAI), which is the value of TT (ng/dL) divided by SHBG (nmol/L) and the ratio of TT to estradiol. These were used to determine the approximate levels of circulating free testosterone and aromatase activity (18, 19).

### 2.3 Covariates

As demographic variables in our model, we incorporated age, gender, race, poverty income ratio (PIR), body mass index (BMI, kg/m^2^), educational level, and diabetes. Serum cotinine (ng/mL), a biomarker indicating exposure to tobacco smoke, was also incorporated. Considering the variations in sex hormone concentrations across different seasons and blood sampling times, we included variables for the six-month time period and venipuncture timing (morning, afternoon, and evening) as covariates (20). Furthermore, we divided BMI into three categories: normal weight (<25 kg/m^2^), overweight (≥25 kg/m^2^-<30 kg/m^2^) and obese (≥30 kg/m^2^).

To account for the differing conditions between children and adolescents, we categorized individuals aged 6–11 years and 12–19 years as children and adolescents, respectively, based on prior research (21, 22). However, categorizing participants aged 6–19 solely based on age into subgroups of children and adolescents may conflate prepubescent and adolescent individuals. This amalgamation could lead to aberrantly high or low hormone levels within each subgroup, potentially distorting the association between SII and sex hormones in regression analyses. Furthermore, the impact of SII on sex hormones may vary depending on the pubertal status. To address this issue, we further stratified participants into pubertal and prepubertal groups based on serum sex hormone levels and the onset of menstruation. Males with a TT level of 50 ng/dL or higher and Females with an estradiol level of 20 pg/mL or higher are classified as the pubertal group. All others are categorized as the prepubertal group (21).

### 2.4 Statistical analysis

In this investigation, continuous variables were presented as mean ± standard deviation, and categorical variables were delineated as proportions. Disparities among distinct SII groups (tertiles) were assessed using chi-square tests for categorical variables and t-tests for continuous variables. To scrutinize the association between SII and sex hormones, three models were devised to examine sex hormones as continuous variables. In model 1, no adjustment for covariates was made. Model 2 was adjusted for age and race. Model 3 was adjusted for age, race, PIR, BMI, education level, seasons and time of venipuncture, serum cotinine, diabetes, and pubertal status. The statistical study used the statistical computing and graphing software R (version 4.2.1) and EmpowerStats (version 2.0). Smooth curve fitting was performed simultaneously by adjusting for variables. A threshold effects analysis model was used to investigate the relationship and inflection points between SII and sex hormones. Finally, the same statistical study methods described above were performed for BMI, diabetes, and pubertal status subgroups. P<0.05 was determined to be statistically significant.

## 3 Results

### 3.1 Baseline characteristics of participants

This investigation enrolled a cohort of 3,767 participants, adhering to predefined inclusion and exclusion criteria. The clinical characteristics of the participants, stratified by gender, are comprehensively outlined in **Table 1**. The mean age of the participants was 12.32 ± 3.95 years, with males constituting 50.54% and females comprising 49.46% of the cohort. The racial/ethnic distribution was as follows: 24.56% Mexican Americans, 12.42% other Hispanic Americans, 26.28% non-Hispanic whites, 21.64% non-Hispanic black people, and 15.10% other racial/ethnic groups. 57.98% male and 31.51% female reached pubertal status.

**Table 1:** Baseline characteristics of participants.

	Male (N=1904)	Female (N=1863)	P-value
**Age**	12.33 ± 3.96	12.31 ± 3.95	0.899
**Race/Ethnicity (%)**			0.061
Mexican American	435 (22.85%)	490 (26.30%)	
Other Hispanic	230 (12.08%)	238 (12.78%)	
Non-Hispanic White	531 (27.89%)	459 (24.64%)	
Non-Hispanic Black	419 (22.01%)	396 (21.26%)	
Other Race	289 (15.18%)	280 (15.03%)	
**PIR**	2.00 ± 1.51	1.94 ± 1.49	0.240
**BMI (kg/m2)**			0.057
Normal weight	1151 (60.96%)	1086 (58.93%)	
Overweight	314 (16.63%)	362 (19.64%)	
Obese	423 (22.40%)	395 (21.43%)	
**Education level (%)**			0.705
Less than high school	1737 (91.81%)	1695 (91.33%)	
High school or GED	101 (5.34%)	108 (5.82%)	
Above high school	53 (2.80%)	53 (2.86%)	
Unknown	1 (0.05%)	0 (0.00%)	
**Six-month time period**			0.054
November 1 through April 30	952 (50.00%)	873 (46.86%)	
November 1 through April 30	952 (50.00%)	990 (53.14%)	
**Time of venipuncture**			0.293
Morning	806 (42.33%)	821 (44.07%)	
Afternoon	712 (37.39%)	651 (34.94%)	
Evening	386 (20.27%)	391 (20.99%)	
**Diabetes (%)**			0.409
Yes	6 (0.32%)	9 (0.49%)	
No	1890 (99.68%)	1840 (99.51%)	
**Pubertal status**			<0.001
Pubertal	1104 (57.98%)	587 (31.51%)	
Prepubertal	800 (42.02%)	1276 (68.49%)	
**Serum cotinine (ng/mL)**	6.57 ± 37.53	3.10 ± 24.19	0.205
**SII**	407.10 ± 228.57	443.95 ± 231.94	<0.001
**Total testosterone (ng/dL)**	220.69 ± 239.52	18.11 ± 15.84	<0.001
**Estradiol (pg/mL)**	11.92 ± 11.66	59.46 ± 331.64	<0.001
**SHBG (nmol/L)**	65.58 ± 47.27	71.26 ± 47.82	<0.001
**Free androgen index**	7.23 ± 8.42	0.42 ± 0.49	<0.001
**Ratio of TT to estradiol**	13.95 ± 14.13	0.98 ± 1.23	<0.001

PIR, poverty income ratio; BMI, body mass index; GED, general educational development; SII, systemic immune-inflammation index; SHBG, sex hormone-binding globulin; TT, total testosterone.

Compared to females, males exhibit relatively higher levels of TT, FAI, and the ratio of TT to estradiol. However, they show relatively lower levels of SII, E2, and SHBG. Moreover, no statistically significant differences were observed in age, PIR, BMI, education level, six-month time period, time of venipuncture, diabetes, and serum cotinine (P > 0.05).

To further characterize our study population, we categorized participants by gender and age groups in **Supplementary Table 1**. Children had lower levels of SII, TT, E2, FAI, and a lower TT to estradiol ratio compared to adolescents, while SHBG levels were higher in children than in adolescents.

### 3.2 Association between SII and sex hormone

Since no apparent effect values were seen, we used SII/100 to enhance the effect values. The results of the multivariate regression analysis between SII/100 and sex hormones, segregated by gender, are presented in **Table 2**. Initially, we assessed the association between SII and sex hormones in males. Within the fully adjusted model, a statistically significant negative correlation was observed between SII and SHBG (β= -0.70, 95% CI: -1.34, -0.06, P=0.0312). With each unit increase in SII, SHBG decreases by 0.7nmol/ml, indicating that higher SII scores correspond to lower SHBG levels. Subsequent analyses were conducted by categorizing SII into tertiles. However, no statistically significant correlation was found (P>0.05). Moreover, in exploring the relationship between SII and other sex hormones, encompassing TT, estradiol, FAI, and the Ratio of TT to estradiol, no statistically significant associations were identified (all P>0.05).

**Table 2:** Association between systemic immune-inflammation index with sex hormone in male and female aged 6–19.

SII/100	β^1^ (95% CI^2^), P value					
	Male			Female		
	Model 1^3^	Model 2^4^	Model 3^5^	Model 1^3^	Model 2^4^	Model 3^5^
Total testosterone (ng/dL)						
Continuous	-3.19 (-7.90, 1.52) 0.1842	-6.08 (-8.74, -3.41) <0.0001	-2.32 (-4.90, 0.27) 0.0788	0.97 (0.66, 1.28) <0.0001	-0.03 (-0.28, 0.22) 0.8016	-0.31 (-0.52, -0.09) 0.0051
Tertile 1	Reference	Reference	Reference	Reference	Reference	Reference
Tertile 2	7.57 (-18.08, 33.22) 0.5631	1.74 (-12.70, 16.18) 0.8134	15.30 (1.66, 28.94) 0.0281	2.06 (0.26, 3.87) 0.0254	-0.78 (-2.18, 0.62) 0.2753	-0.41 (-1.59, 0.77) 0.4929
Tertile 3	-13.42 (-40.01, 13.17) 0.3227	-27.03 (-42.09, -11.97) 0.0004	-2.12 (-16.55, 12.31) 0.7735	4.81 (3.06, 6.57) <0.0001	-0.71 (-2.12, 0.70) 0.3227	-1.51 (-2.71, -0.30) 0.0141
P for trend	-3.90 (-10.98, 3.17) 0.2795	-7.55 (-11.56, -3.55) 0.0002	-1.09 (-4.92, 2.75) 0.5797	1.27 (0.81, 1.73) <0.0001	-0.16 (-0.52, 0.21) 0.4048	-0.41 (-0.73, -0.10) 0.0104
Estradiol (pg/mL)						
Continuous	0.11 (-0.12, 0.34) 0.3472	-0.07 (-0.20, 0.06) 0.2730	-0.05 (-0.18, 0.08) 0.4410	12.88 (6.41, 19.35) <0.0001	8.26 (1.53, 14.99) 0.0162	0.53 (-0.51, 1.57) 0.3158
Tertile 1	Reference	Reference	Reference	Reference	Reference	Reference
Tertile 2	0.64 (-0.61, 1.89) 0.3168	0.38 (-0.30, 1.07) 0.2723	0.40 (-0.29, 1.08) 0.2577	8.91 (-29.10, 46.93) 0.6458	-5.24 (-43.42, 32.94) 0.7878	4.96 (-0.75, 10.67) 0.0886
Tertile 3	0.92 (-0.37, 2.22) 0.1624	0.09 (-0.62, 0.80) 0.8050	0.17 (-0.55, 0.89) 0.6468	47.14 (10.19, 84.08) 0.0125	20.84 (-17.47, 59.14) 0.2865	6.20 (0.37, 12.03) 0.0374
P for trend	0.24 (-0.11, 0.58) 0.1740	0.01 (-0.18, 0.20) 0.8846	0.03 (-0.16, 0.23) 0.7253	13.12 (3.47, 22.78) 0.0078	6.41 (-3.60, 16.41) 0.2096	1.49 (-0.04, 3.02) 0.0560
SHBG (nmol/L)						
Continuous	-2.16 (-3.09, -1.24) <0.0001	-1.62 (-2.28, -0.97) <0.0001	-0.70 (-1.34, -0.06) 0.0312	-4.15 (-5.07, -3.24) <0.0001	-3.21 (-4.10, -2.32) <0.0001	-2.08 (-2.94, -1.21) <0.0001
Tertile 1	Reference	Reference	Reference	Reference	Reference	Reference
Tertile 2	-9.48 (-14.50, -4.45) 0.0002	-6.95 (-10.49, -3.41) 0.0001	-2.96 (-6.32, 0.40) 0.0846	-16.71 (-22.07, -11.35) <0.0001	-14.11 (-19.16, -9.07) <0.0001	-11.87 (-16.64, -7.11) <0.0001
Tertile 3	-14.59 (-19.80, -9.38) <0.0001	-11.04 (-14.73, -7.35) <0.0001	-3.35 (-6.91, 0.20) 0.0648	-25.82 (-31.03, -20.61) <0.0001	-20.01 (-25.07, -14.94) <0.0001	-13.40 (-18.27, -8.53) <0.0001
P for trend	-3.79 (-5.18, -2.41) <0.0001	-2.87 (-3.85, -1.88) <0.0001	-0.84 (-1.79, 0.10) 0.0816	-6.46 (-7.82, -5.10) <0.0001	-4.91 (-6.24, -3.59) <0.0001	-3.15 (-4.43, -1.87) <0.0001
Free androgen index						
Continuous	0.15 (-0.02, 0.31) 0.0840	-0.03 (-0.11, 0.06) 0.5689	-0.03 (-0.12, 0.06) 0.5196	0.05 (0.04, 0.06) <0.0001	0.02 (0.01, 0.03) <0.0001	0.00 (-0.00, 0.01) 0.4037
Tertile 1	Reference	Reference	Reference	Reference	Reference	Reference
Tertile 2	0.91 (0.01, 1.81) 0.0477	0.56 (0.08, 1.04) 0.0227	0.51 (0.04, 0.98) 0.0349	0.13 (0.07, 0.18) <0.0001	0.06 (0.01, 0.11) 0.0165	0.03 (-0.01, 0.08) 0.1426
Tertile 3	1.08 (0.15, 2.02) 0.0231	0.24 (-0.26, 0.74) 0.3540	0.17 (-0.33, 0.67) 0.4991	0.28 (0.22, 0.33) <0.0001	0.14 (0.09, 0.19) <0.0001	0.05 (0.01, 0.10) 0.0269
P for trend	0.28 (0.03, 0.52) 0.0294	0.05 (-0.08, 0.18) 0.4633	0.03 (-0.10, 0.17) 0.6403	0.07 (0.06, 0.09) <0.0001	0.04 (0.02, 0.05) <0.0001	0.01 (0.00, 0.02) 0.0347
Ratio of TT to estradiol						
Continuous	-0.27 (-0.54, 0.01) 0.0609	-0.23 (-0.45, -0.02) 0.0341	0.05 (-0.15, 0.26) 0.6180	-0.06 (-0.08, -0.03) <0.0001	-0.03 (-0.06, -0.01) 0.0077	-0.03 (-0.06, -0.01) 0.0096
Tertile 1	Reference	Reference	Reference	Reference	Reference	Reference
Tertile 2	-0.49 (-2.01, 1.02) 0.5225	-0.75 (-1.91, 0.42) 0.2095	0.21 (-0.87, 1.28) 0.7065	-0.08 (-0.22, 0.06) 0.2725	-0.02 (-0.15, 0.12) 0.8097	-0.06 (-0.20, 0.08) 0.4315
Tertile 3	-1.68 (-3.24, -0.11) 0.0360	-1.57 (-2.79, -0.36) 0.0112	0.23 (-0.91, 1.37) 0.6881	-0.32 (-0.45, -0.18) <0.0001	-0.18 (-0.31, -0.04) 0.0092	-0.19 (-0.33, -0.04) 0.0106
P for trend	-0.45 (-0.87, -0.03) 0.0340	-0.42 (-0.74, -0.09) 0.0117	0.06 (-0.24, 0.36) 0.7050	-0.09 (-0.12, -0.05) <0.0001	-0.05 (-0.09, -0.02) 0.0045	-0.05 (-0.09, -0.01) 0.0080

^1^β, effect sizes.

^2^95% CI, 95% confidence interval.

^3^Model 1, no covariates were adjusted.

^4^Model 2, adjusted for age and race.

^5^Model 3, adjusted for age, race, poverty income ratio, body mass index, educational level, six-month time period, time of venipuncture, diabetes, pubertal status and serum cotinine.

In the analysis of the association between female SII and sex hormones, our fully adjusted model revealed a significant negative correlation when treating SII as a continuous variable. Specifically, SII exhibited significant negative correlations with TT (β=-0.31, 95% CI: -0.52, -0.09, p=0.0051), SHBG (β=-2.08, 95% CI: -2.94, -1.21, p<0.0001), and Ratio of TT to estradiol (β=-0.03, 95% CI: -0.06, -0.01, p=0.0096). When SII was converted into three strata, the significance with TT (β=-0.41, 95% CI: -0.73, -0.10, p=0.0104), SHBG (β=-3.15, 95% CI: -4.43, -1.87, p<0.0001), and ratio of TT to estradiol (β=-0.05, 95% CI: -0.09, -0.01, p=0.0080) remained unchanged. Furthermore, we found that as a continuous variable, SII had no statistical significance with FAI (P>0.05). Still, after tertile, SII was positively correlated with FAI (β=0.01, 95% CI: 0.00, -0.02, p=0.0347).

To assess whether the association between SII and sex hormones was consistent across the entire population and to identify potential variations within distinct population subgroups, we conducted meticulous subgroup analyses and interaction tests based on diabetes status, body mass index, and puberty status. Our results indicated that in males, SII did not have a significant correlation with these subgroups (**Table 3**). Conversely, we observed a more consistent association within female subgroups. Notably, **Table 4** highlights significant interactions between BMI and FAI, as well as between pubertal status and estradiol (interaction P <0.05).

**Table 3:** Subgroup analysis for the association between SII and sex hormones in male aged 6–19.

SII/100	Total testosterone		Estradiol		SHBG		Free androgen index		Ratio of TT to estradiol	
	β^1^ (95% CI^2^) P value	P for interaction	β^1^ (95% CI^2^) P value	P for interaction	β^1^ (95% CI^2^) P value	P for interaction	β^1^ (95% CI^2^) P value	P for interaction	β^1^ (95% CI^2^) P value	P for interaction
**Diabetes**		1.000		1.0000		1.0000		1.0000		1.0000
Yes	41.20 (-95.69, 178.08) 0.5553		0.17 (-6.70, 7.03) 0.9617		3.33 (-30.30, 36.96) 0.8460		-0.76 (-5.52, 4.00) 0.7544		2.37 (-8.40, 13.13) 0.6664	
No	-2.31 (-4.90, 0.29) 0.0815		-0.05 (-0.18, 0.08) 0.4514		-0.67 (-1.31, -0.03) 0.0395		-0.03 (-0.12, 0.06) 0.5559		0.05 (-0.15, 0.26) 0.6067	
**BMI**		0.8384		0.9131		0.5227		0.1159		0.3824
BMI < 25 kg/m^2^	-2.90 (-6.08, 0.28) 0.0736		-0.05 (-0.22, 0.11) 0.5172		-0.64 (-1.40, 0.13) 0.1058		0.04 (-0.07, 0.15) 0.4491		0.05 (-0.20, 0.31) 0.6703	
BMI ≥ 25 kg/m^2^, < 30 kg/m^2^	-1.03 (-7.54, 5.49) 0.7578		-0.03 (-0.37, 0.30) 0.8388		0.30 (-1.27, 1.88) 0.7061		-0.17 (-0.40, 0.06) 0.1408		0.13 (-0.38, 0.64) 0.6215	
BMI ≥ 30 kg/m^2^	-4.20 (-10.27, 1.88) 0.1760		-0.08 (-0.39, 0.23) 0.6138		-1.48 (-2.95, -0.01) 0.0485		-0.10 (-0.32, 0.11) 0.3422		-0.23 (-0.70, 0.25) 0.3566	
**Pubertal status**		0.3417		0.8887		0.1216		0.7079		0.4908
Pubertal	-2.43 (-5.69, 0.82) 0.1428		-0.02 (-0.19, 0.15) 0.8145		-0.17 (-0.99, 0.65) 0.6870		0.03 (-0.08, 0.15) 0.5613		0.12 (-0.15, 0.38) 0.3911	
Prepubertal	-0.06 (-3.81, 3.69) 0.9751		-0.00 (-0.20, 0.19) 0.9833		-1.14 (-2.08, -0.20) 0.0178		0.00 (-0.13, 0.13) 0.9878		-0.02 (-0.33, 0.28) 0.8770	

^1^β, effect sizes.

^2^95% CI, 95% confidence interval.

**Table 4:** Subgroup analysis for the association between SII and sex hormones in female aged 6–19.

SII/100	Total testosterone		Estradiol		SHBG		Free androgen index		Ratio of TT to estradiol	
	β^1^ (95% CI^2^) P value	P for interaction	β^1^ (95% CI^2^) P value	P for interaction	β^1^ (95% CI^2^) P value	P for interaction	β^1^ (95% CI^2^) P value	P for interaction	β^1^ (95% CI^2^) P value	P for interaction
**Diabetes**		1.0000		1.0000		1.0000		1.0000		1.0000
Yes	2.05 (-5.86, 9.97) 0.6112		7.79 (-30.80, 46.38) 0.6924		-8.48 (-40.74, 23.79) 0.6067		0.13 (-0.18, 0.43) 0.4086		-0.06 (-1.00, 0.88) 0.8968	
No	-0.32 (-0.54, -0.11) 0.0029		0.53 (-0.51, 1.57) 0.3147		-2.11 (-2.98, -1.24) <0.0001		0.00 (-0.00, 0.01) 0.3851		-0.03 (-0.06, -0.01) 0.0090	
**BMI**		0.2286		0.5828		0.3671		0.0326		0.2310
BMI < 25 kg/m^2^	-0.23 (-0.52, 0.07) 0.1312		0.74 (-0.70, 2.17) 0.3135		-2.80 (-3.99, -1.60) <0.0001		0.01 (-0.00, 0.02) 0.0856		-0.03 (-0.06, 0.01) 0.1456	
BMI ≥ 25 kg/m^2^, < 30 kg/m^2^	-0.28 (-0.78, 0.22) 0.2682		1.16 (-1.26, 3.59) 0.3477		-0.82 (-2.83, 1.20) 0.4264		-0.01 (-0.02, 0.01) 0.4916		-0.02 (-0.07, 0.04) 0.5865	
BMI ≥ 30 kg/m^2^	-0.56 (-1.02, -0.11) 0.0141		-0.12 (-2.31, 2.07) 0.9157		-2.13 (-3.95, -0.31) 0.0217		-0.01 (-0.03, 0.01) 0.2347		-0.07 (-0.12, -0.02) 0.0115	
**Pubertal status**		0.3864		0.0194		0.9631		0.2536		0.1807
Pubertal	-0.20 (-0.53, 0.13) 0.2427		2.17 (0.58, 3.76) 0.0075		-2.06 (-3.41, -0.71) 0.0028		0.01 (-0.01, 0.02) 0.2399		-0.01 (-0.04, 0.03) 0.7481	
Prepubertal	-0.39 (-0.67, -0.10) 0.0073		-0.28 (-1.64, 1.07) 0.6842		-2.02 (-3.17, -0.87) 0.0006		-0.00 (-0.01, 0.01) 0.7254		-0.04 (-0.07, -0.01) 0.0162	

^1^β, effect sizes.

^2^95% CI, 95% confidence interval.

**Figure 2:**
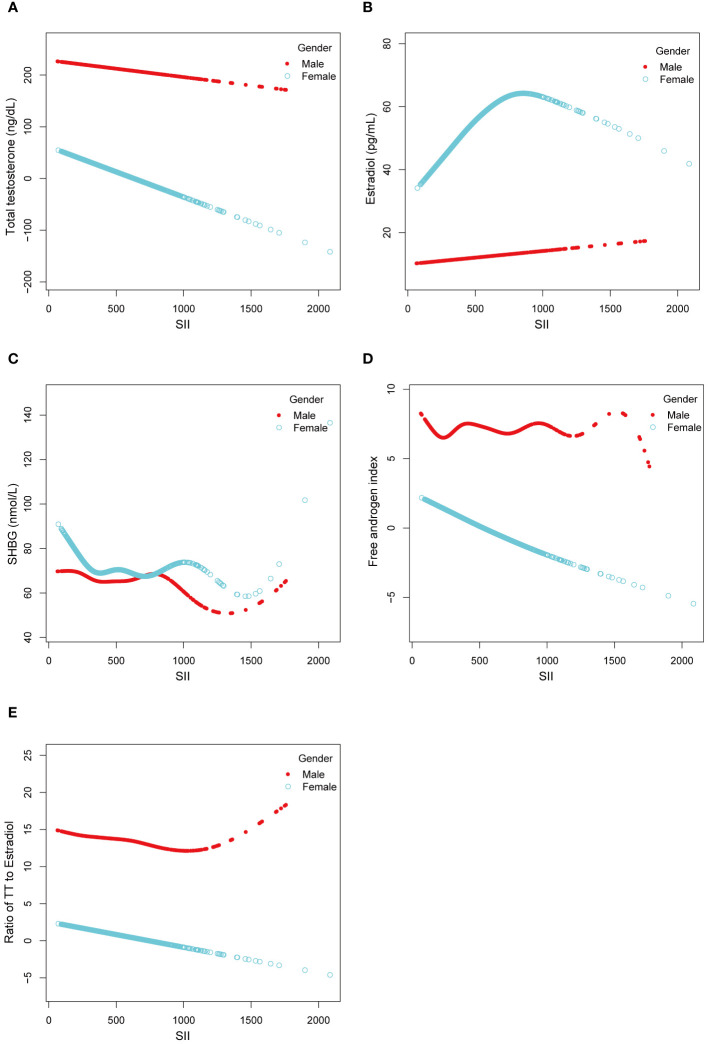
Linear and Non-linear relationship between SII and sex hormones among male and female aged 6-19. **(A)** TT and SII; **(B)** Estradiol and SII; **(C)** SHBG and SII; **(D)** FAI and SII; **(E)** Ratio of TT to Estradiol and SII.

We utilized a smoothing curve fitting method to illustrate the nonlinear relationship between SII and sex hormones, as shown in **Figure 2**. The variables adjusted in this analysis included age, race, PIR, BMI, education level, blood draw season and time, serum cotinine, diabetes, and puberty status. Utilizing a two-stage linear regression model, we unveiled a non-linear association between SII and sex hormones. Intriguingly, within the female population, an inverted U-shaped curve was discerned in the non-linear relationship between SII and estradiol, which appeared at 748.09 (**Table 5**).

**Table 5:** Threshold effect analysis of SII on estradiol (pg/mL) using two-piecewise linear regression model.

Estradiol (pg/mL)	Adjusted β (95% CI) P value
SII	
Inflection point	778.42
SII < 778.42	0.03 (0.02, 0.04) <0.0001
SII > 778.42	-0.02 (-0.04, -0.00) 0.0426
Log likelihood ratio	<0.001
Male	
Inflection point	595.79
SII < 595.79	0.00 (-0.00, 0.00) 0.3410
SII > 595.79	-0.00 (-0.00, 0.00) 0.0751
Log likelihood ratio	0.096
Female	
Inflection point	748.09
SII < 748.09	0.02 (0.01, 0.04) 0.0013
SII > 748.09	-0.04 (-0.06, -0.01) 0.0043
Log likelihood ratio	<0.001

Adjusted for age, race, poverty income ratio, body mass index, educational level, six-month time period, time of venipuncture, diabetes, pubertal status and serum cotinine.

SII, systemic immune-inflammation index.

## 4 Discussions

Our cross-sectional study involving 3767 children and adolescents identified significant correlations between the SII and specific sex hormones, even after adjusting for potential confounding factors. Notably, a substantial negative correlation emerged between SII and SHBG in males. In females, SII demonstrated significant negative associations with TT, SHBG, and the ratio of TT to estradiol, accompanied by a positive correlation with the FAI. Subgroup analysis and interaction tests unveiled variations among different populations, underscoring the nuanced nature of these relationships. Intriguingly, the relationship between SII and estradiol in females followed an inverted U-shaped curve, with an inflection point at 748.09 (1000 cells/ml). This finding suggests a complex interplay between systemic immune inflammation and sex hormones in this population.

This is the first study to evaluate the association between SII and sex hormones in children and adolescents. Previous clinical investigations have suggested a link between inflammation indices and sex hormones. For instance, Osmancevic et al. examined a community sample of 3041 men aged 45–84 years. They found a negative correlation between hsCRP levels, a biomarker for detecting chronic inflammation, and testosterone and SHBG levels (23). Similarly, Ma et al. using a similar NHANES participant, investigated 1382 adolescents and children aged 6–19 years and found a negative correlation between the Dietary Inflammatory Index (DII) and TT (P = 0.01) and FAI (P = 0.02) in female adolescents. In prepubescent males, DII exhibited a positive correlation with SHBG, while in prepubescent females, it demonstrated a negative association with FAI (24).. Wei et al. through a survey of 9372 adults, found a significant negative correlation between SII and SHBG in both male and female populations (25). These studies are consistent with our results, suggesting that inflammation-related factors may be associated with sex hormone levels. However, investigations into the relationship between immune-inflammatory factors and diverse sex hormone levels, particularly in the context of children and adolescents, are notably scarce in the current studies. Our study incorporated various confounding factors, analyzed male and female adolescents separately, explored the linear and non-linear associations between SII and sex hormones, conducted stratified analysis, and discovered the potential impact of pubertal status and BMI on the association between SII and sex hormone concentrations.

The gender-specific serum levels and metabolism of sex hormones largely account for the higher incidence of autoimmune diseases in females than in males (26). Moreover, numerous studies suggest that androgens are anti-inflammatory, while estrogens are pro-inflammatory (27–29). Studies have found that pro-inflammatory cytokines (TNF-α, IL-1β, IL-6) can increase the expression of aromatase, an enzyme that converts testosterone into estrogen, thereby accelerating the metabolic conversion of androgens to estrogens and reducing the amount of available testosterone in the body (30, 31). Our study results showed a significant negative correlation between SII and the ratio of TT to estradiol. We speculate this is mainly due to a significant decrease in testosterone levels. However, it’s important to note that the ratio of TT to estradiol may not accurately predict aromatase activity, as it is influenced by other factors, such as metabolic syndrome (32), introducing uncertainty to this result. In addition, under inflammatory conditions, these inflammatory factors also stimulate macrophages to produce pro-inflammatory cytokines, which inhibit Leydig cells. Macrophages also have Reactive Oxygen Species (ROS), hindering Leydig cell function and decreasing testosterone synthesis (33, 34). In animal experiments, systemic inflammation caused by bacterial infection in the mother during early embryonic development interferes with the migration and synapse formation of Gonadotropin-Releasing Hormone (GnRH) neurons. This interference affects the development and function of the Hypothalamic-Pituitary-Gonadal (HPG) axis in male rat offspring, resulting in reduced gonadotropin and sex steroid levels and gonadal structural damage (35). Klaudia Barabás et al. demonstrated that in females, inflammation inhibits the secretion of GnRH and Luteinizing Hormone (LH), leading to ovulation disorders and infertility (36). Furthermore, research indicates that Visceral Adipose Tissue (VAT) is a source of endogenous estrogen and pro-inflammatory cytokines, potentially reducing the synthesis of SHBG and decreasing serum SHBG levels (37). This finding aligns with our research results, indicating a negative correlation between SII and SHBG. However, it’s essential to note that no definitive research is currently establishing the relationship between SII and FAI. MaryFran R Sowers’ research suggests that androgens can influence the inflammatory response through various pathways. High FAI and low SHBG may reflect elevated levels of androgens (38).

Our findings bear significant implications for clinical application and future research. Firstly, our study utilized a nationally representative sample, reflecting the ethnically and gender-diverse population of children and adolescents in the United States. The substantial sample size enabled subgroup analyses, contributing to the robustness and generalizability of our results. Moreover, our findings suggest that a high SII may precede potential risks of sex hormone disorders, providing a basis for preventive strategies against such disorders through SII monitoring. However, our study has limitations. Firstly, we cannot establish definitive causal relationships due to the cross-sectional study design. Secondly, although we adjusted for several relevant confounding factors, we could only incorporate some covariates, thus limiting the attainment of entirely accurate results. Finally, we acknowledge that the presence of inflammatory diseases or infections could elevate inflammatory levels and confound our results. However, data on such conditions were not available and should be considered a limitation of this study.

## 5 Conclusions

Our findings suggest that SII may be an independent risk factor for changes in sex hormones in both male and female children and adolescents. More prospective and experimental studies should be conducted to validate our results and elucidate the underlying molecular pathways.

## Data availability statement

The datasets presented in this study can be found in online repositories. The names of the repository/repositories and accession number(s) can be found below: https://www.cdc.gov/nchs/nhanes/.

## Ethics statement

The studies involving humans were approved by NCHS Ethics Review Board (ERB). The studies were conducted in accordance with the local legislation and institutional requirements. Written informed consent for participation in this study was provided by the participants’ legal guardians/next of kin.

## Author contributions

ZG: Writing – review & editing, Writing – original draft, Supervision, Software, Project administration, Conceptualization. KL: Writing – review & editing, Writing – original draft, Visualization, Validation, Investigation, Formal analysis, Conceptualization.
