# Supplementary material for: Association between systemic immunity-inflammation index and sex hormones in children and adolescents aged 6–19
Source: Front Endocrinol (Lausanne). 2024 Jun 13;15:1355738. doi: 10.3389/fendo.2024.1355738 (PMC11208618; doi:10.3389/fendo.2024.1355738)
Supplement: Supplementary file 1 [file Table_1.docx]

**Supplementary Table 1**. Characteristics of 6-19-year-old children and adolescents with serum sex hormones and systemic immune-inflammation index (SII) in NHANES 2013-2016

|  | Male (N=1904) | | Female (N=1863) | |
| --- | --- | --- | --- | --- |
|  | Children (N=852) | Adolescents (N=1052) | Children (N=838) | Adolescents (N=1025) |
| **AGE** | 8.54 ± 1.70 | 15.39 ± 2.23 | 8.57 ± 1.75 | 15.37 ± 2.23 |
| **Race/Ethnicity (%)** |  |  |  |  |
| Mexican American | 191 (22.42%) | 244 (23.19%) | 232 (27.68%) | 258 (25.17%) |
| Other Hispanic | 115 (13.50%) | 115 (10.93%) | 103 (12.29%) | 135 (13.17%) |
| Non-Hispanic White | 230 (27.00%) | 301 (28.61%) | 210 (25.06%) | 249 (24.29%) |
| Non-Hispanic Black | 190 (22.30%) | 229 (21.77%) | 185 (22.08%) | 211 (20.59%) |
| Other Race | 126 (14.79%) | 163 (15.49%) | 108 (12.89%) | 172 (16.78%) |
| **PIR** | 2.02 ± 1.54 | 1.99 ± 1.49 | 1.90 ± 1.53 | 1.97 ± 1.46 |
| **BMI (kg/m2)** |  |  |  |  |
| Normal weight | 518 (61.01%) | 633 (60.92%) | 511 (61.20%) | 575 (57.04%) |
| Overweight | 135 (15.90%) | 179 (17.23%) | 157 (18.80%) | 205 (20.34%) |
| Obese | 196 (23.09%) | 227 (21.85%) | 167 (20.00%) | 228 (22.62%) |
| **Education level (%)** |  |  |  |  |
| Less than high school | 850 (100.00%) | 887 (85.21%) | 835 (100.00%) | 860 (84.23%) |
| High school or GED | - | 101 | - | 108 |
| Above high school | - | 53 | - | 53 |
| Unknown | - | 1 | - | - |
| **Six-month time period** |  |  |  |  |
| November 1 through April 30 | 399 (46.83%) | 553 (52.57%) | 388 (46.3%) | 485 (47.32%) |
| November 1 through April 30 | 453 (53.17%) | 499 (47.43%) | 450 (53.7%) | 540 (52.68%) |
| **Time of venipuncture** |  |  |  |  |
| Morning | 321 (37.68%) | 485 (46.1%) | 329 (39.26%) | 492 (46.95%) |
| Afternoon | 336 (39.44%) | 376 (35.74%) | 323 (38.54%) | 328 (31.30%) |
| Evening | 195 (22.89%) | 191 (18.16%) | 186 (22.20%) | 228 (21.76%) |
| **Diabetes (%)** |  |  |  |  |
| Yes | 3 (0.35%) | 3 (0.29%) | 2 (0.24%) | 7 (0.67%) |
| No | 846 (99.65%) | 1044 (99.71%) | 832 (99.76%) | 1008 (99.33%) |
| **Pubertal status** |  |  |  |  |
| Pubertal | 90 (10.56%) | 1014 (96.39%) | 45 (5.37%) | 542 (52.88%) |
| Prepubertal | 762 (89.44%) | 38 (3.61%) | 793 (94.63%) | 483 (47.12%) |
| **Serum cotinine (ng/mL)** | 0.34 ± 1.04 | 11.60 ± 49.90 | 0.32 ± 0.92 | 5.36 ± 32.41 |
| **SII** | 402.50 ± 222.98 | 410.83 ± 233.04 | 385.78 ± 189.50 | 491.51 ± 251.87 |
| **Total testosterone (ng/dL)** | 19.17 ± 60.42 | 383.90 ± 203.31 | 7.75 ± 7.74 | 26.57 ± 15.74 |
| **Estradiol (pg/mL)** | 2.54 ± 1.81 | 19.51 ± 10.69 | 13.56 ± 30.68 | 96.98 ± 442.82 |
| **SHBG (nmol/L)** | 98.63 ± 47.96 | 38.81 ± 24.10 | 83.35 ± 43.54 | 61.38 ± 48.91 |
| **Free androgen index** | 0.40 ± 1.59 | 12.76 ± 7.61 | 0.16 ± 0.24 | 0.63 ± 0.54 |
| **Ratio of TT to Estradiol** | 4.60 ± 9.92 | 21.53 ± 12.38 | 1.31 ± 1.01 | 0.72 ± 1.32 |

PIR, poverty income ratio; BMI, body mass index; GED, general educational development; SII, systemic immune-inflammation index; SHBG, sex hormone-binding globulin; TT, total testosterone.
